# Documenting medico-legal evidence in Kenya: Potential strategies for improvement

**DOI:** 10.1186/1753-6561-9-S4-A2

**Published:** 2015-07-07

**Authors:** Carol Ajema, Wanjiru Mukoma, Ronald Kotut, Reuben Mulwa

**Affiliations:** 1 LVCT Health, P.O. Box 19835-00202, KNH Nairobi, Kenya

## Background

Sexual violence (SV) remains a global public health problem and a violation of multiple human rights. It can negatively impact on the short- and long-term physical, social and mental health of survivors and is associated with many adverse health outcomes [1-3]. It is assumed that some of the negative outcomes of SV can be addressed through the provision of appropriate post-rape care services [4]. The latter include the collection and documentation of medico-legal evidence, which is central to the success of prosecution efforts and positive legal outcomes. In Kenya, where one in five women has experienced SV [5] and where the criminal justice system relies heavily on medico-legal evidence collected by health care providers, significant gaps exist in how medico-legal evidence is collected and recorded by providers [6]. One of the main gaps identified is a lack of understanding among health providers and police of the national documentation forms to be used in capturing survivor data. In response to this barrier, this study aimed to improve the documentation of medico-legal evidence in Kenya in order to facilitate improved health and legal outcomes for SV survivors.

## Materials and methods

### Study design

This was an operations research study that used a pre- and post-intervention design with a comparison arm to test the hypothesis that medico-legal evidence documentation in the intervention site would show greater improvement than would be the case in the comparison site by the end of the intervention period.

The study was carried out in two districts in Kenya - Kitui and Rachuonyo - with Kitui District Hospital and Kitui Police Station acting as the intervention sites, while Rachuonyo District Hospital and Rachuonyo Police Station served as the comparison sites.

### Intervention

The intervention was conducted over a 7-month period in 2011. It comprised three main components: training of health providers, police officers and prosecutors; the introduction of a locally-assembled rape kit into the health facility; and the revision of the national clinical algorithm for guiding providers in the collection and documentation of medico-legal evidence at different service delivery points.

The training of health providers (doctors, nurses, clinical officers, and laboratory technicians), police (officers station at the Gender Desk and Crime Unit), and prosecutors was conducted by a team of resource persons drawn from the Division of Reproductive Health, the laboratory of the Government Chemist, the Director of Public Prosecution's Office, and LVCT Health post-rape care program officers. The training session included modules on forensic examination, utilization of the rape kit, completion and utilization of the national documentation forms (the post-rape care ['PRC'] form (also referred to us Ministry of Health form 363) and the Kenya police medical examination ['P3'] form), and referrals between the police station and health facility. According to the national sexual violence guidelines, both forms must be filled out for each survivor and attached to one another to facilitate any intended legal processes. The P3 form contains sections to be filled in by providers at both health facilities and police stations. The training therefore also aimed to equip police officers and health providers with knowledge of their role in providing post-rape care services, including their joint responsibility in enlightening the survivors about the importance of having the completed forms returned to the police before being forwarded to the prosecution office.

To aid intervention site health providers in proper documentation of medico-legal evidence, the national clinical algorithm chart used for this purpose was revised. The revisions included clarifications on the types of information to be documented by health providers, and the tools to be used at different service delivery points for collecting evidence from survivors. A primary tool used for evidence collection was the locally-assembled, pre-packaged rape kit, which brought together various simple tools (e.g., powder-fee gloves, speculum, seal lock bags, stick swabs, pregnancy testing kit, etc.) in one packet to permit evidence collection from the survivor in one location and by one health provider. Ensuring that evidence collection largely happened in one location was also meant to ease the documentation process.

### Data collection and analysis

Retrospective record reviews of post-rape care forms and P3 forms in health facility and police station files were conducted at baseline and endline. Data from these reviews were entered into EPI info 7 and analyzed through cross tabulations using SPSS version 13, and taking into account the study objectives.

## Results

### Provider completion of P3 forms

**Figure 1:**
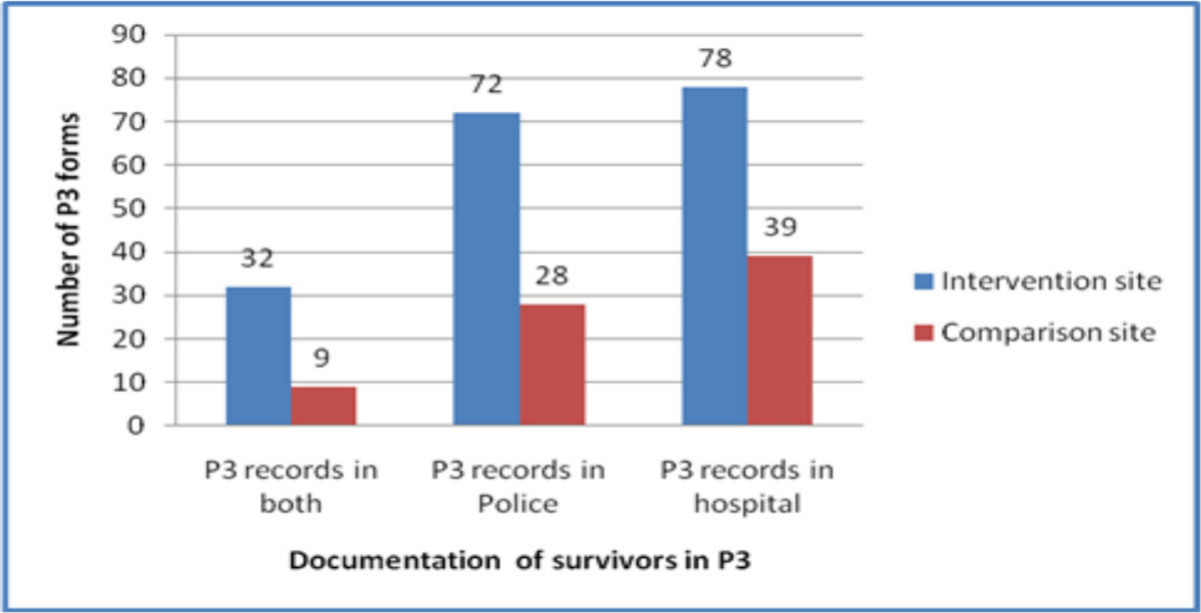


By the endline period, the intervention site was more than three times as likely as the comparison site to have the police station and health facility sections of the P3 forms accurately filled in (Figure 1). It is noteworthy that this seeming improvement in the completion of P3 forms in the intervention site describes the number of forms correctly filled, rather than the proportion of survivors whose forms were correctly filled. The unavailability of data on actual proportions is a limitation of this study, as such information would have provided a more accurate picture of the intervention's effect. A review of the police station sections (of the P3 form) alone, and of the health facility sections alone, also indicated that the intervention site was more likely to complete these sole sections than the comparison site.

### Provider completion of PRC (post-rape care) forms

**Figure 2:**
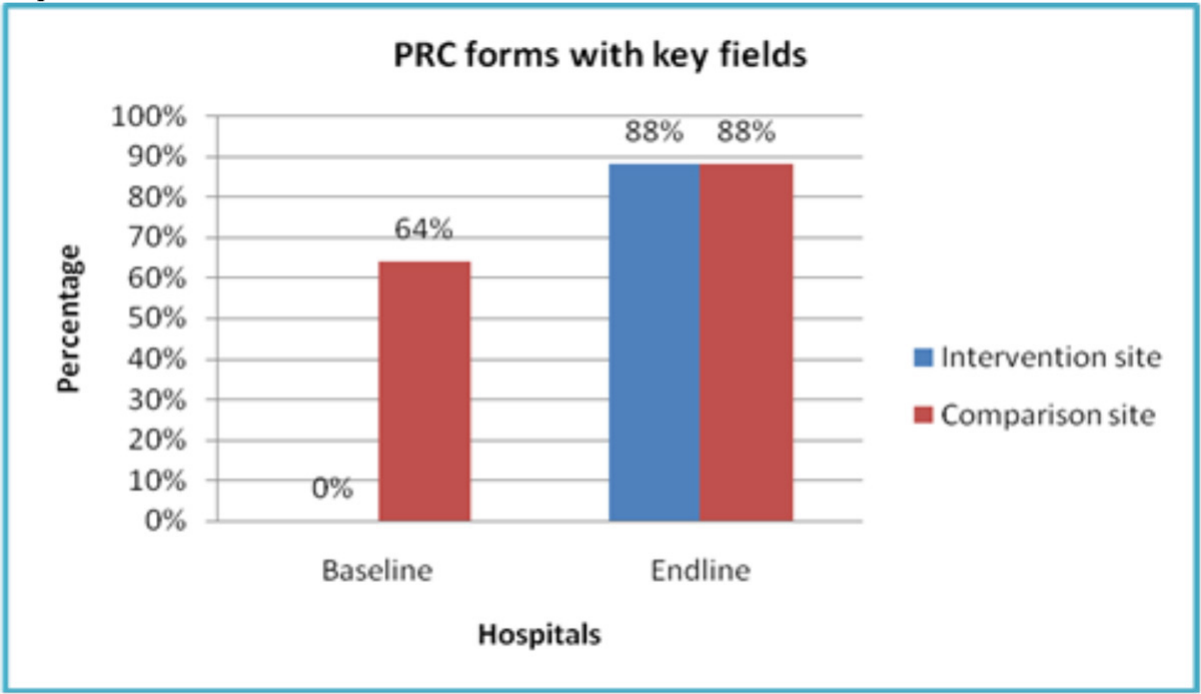


At baseline, the intervention and comparison sites started off on unequal footing, with 64% of SV survivor cases being documented on PRC forms in the comparison health facility site, versus no documentation at all on PRC forms in the intervention health facility site (Figure 2). Earlier provider trainings conducted about two years before the present study at the comparison health facility site explain this discrepancy. Although this earlier training was also offered at the intervention site, policy changes at this site severely limited the cadre of providers that could fill out PRC forms. This policy was abolished during the intervention period, when the health facility administration was sensitized on the revised national post-rape care guidelines, which allow the examining doctor, nurse, or clinical officer to perform this function.

By endline, the proportion of PRC forms that were accurately filled in by health providers in the intervention site rose from 0% to 88%. The comparison site also recorded a slight (but comparatively much lower) improvement in documenting survivor SV cases on the PRC form (from 64% to 88%).

## Conclusions

Findings from this study suggest that multi-sectoral provider training sessions focused on existing national documents for use in the management of reported SV cases, or for the referral of survivors from one sector to the next, can potentially help improve the level of documentation of such cases by providers. This strategy is particularly important in settings where national documents have been developed and are used as medico-legal evidence in SV cases.

Since the completion of this study, Kenya's national post-rape care form and the 2^nd ^edition of the *National Guidelines on Management of Sexual Violence in Kenya *were officially revised, drawing on the study findings, and the multi-sectoral provider training approach described here has been adopted by Kenya's Sexual Offences Act Task force. Further funding was also obtained to explore and strengthen mechanisms for medico-legal evidence across sectors.
